# Knowledge and Awareness About the Basics of Cardiopulmonary Resuscitation in the Saudi Population

**DOI:** 10.7759/cureus.77950

**Published:** 2025-01-25

**Authors:** Shahad Alshammari​​, Adel H Alshammari, Manal Aldosari, Hejab A Aldawsari, Afnan Almass

**Affiliations:** 1 General Practice, Health Cluster, Hafar Albatin, SAU; 2 General Practice, Health Cluster, Riyadh, SAU; 3 Anesthesiology, Alhabib Medical Group, Riyadh, SAU; 4 Emergency Medicine, Ministry of Health, Riyadh, SAU

**Keywords:** cardiac arrest, cardiopulmonary resuscitation, emergency, knowledge and awareness, training

## Abstract

Introduction

According to the World Health Organization (WHO), cardiovascular diseases are the leading cause of death globally, accounting for approximately 17 million deaths annually, with sudden cardiac arrest (SCA) as a significant contributor to this alarming statistic. SCA, the abrupt loss of heart function, is a critical medical emergency that requires early recognition and immediate cardiopulmonary resuscitation (CPR) for the effective resuscitation of victims. Various studies have shown a low level of knowledge regarding CPR in the community. This study aims to evaluate the knowledge and awareness of CPR among the Saudi population, identify gaps, and propose targeted health interventions, including education campaigns and training programs, to enhance community preparedness in emergencies and improve survival rates for SCA victims.

Methods

A cross-sectional observational study was conducted from May 2023 to November 2023. The study population included citizens and residents aged 18 years and older from the Central, Eastern, and Western regions of Saudi Arabia to ensure a diverse range of cultural and social backgrounds, with a sample size of 4,932 participants. Data were collected using an online, validated, closed-ended, structured questionnaire distributed via social media platforms, specifically WhatsApp and Telegram. Statistical analysis was performed using R version 4.3 (R Foundation for Statistical Computing, Vienna, Austria). Counts and percentages were used to summarize categorical variables. The chi-square test of independence was used to assess associations between categorical variables. The unpaired t-test and Mann-Whitney test were used to compare continuous normal and non-normal variables.

Results

A total of 4932 respondents completed the questionnaire. The gender distribution was predominantly female (76.5%, n = 3775), compared to 23.5% (n = 1157) males. The age distribution was skewed toward the younger population, with 59.1% (n = 2914) aged between 18 and 30 years. The educational level was predominantly university-level (68.8%, n = 3391). The academic specialization was diverse, with the largest group being healthcare practitioners (47.1%, n = 1615). The study found that 44.7% (n = 2203) of respondents had received basic life support (BLS) or first aid training, while 55.3% (n = 2729) had not. Results suggest a trend toward higher knowledge levels. The average knowledge score among the respondents was 7.17 ± 2.37, indicating that respondents answered slightly more than half of the questions correctly. The minimum score in the dataset was 1, suggesting that nearly all respondents had some level of correct answers. The maximum score achieved is 13, indicating the presence of individuals with a comprehensive understanding of the assessed topic. However, there is still room for improvement in knowledge and emergency preparedness among the surveyed group.

Conclusion

The study found a relatively high level of knowledge and awareness regarding CPR effectiveness, with higher levels observed among individuals with advanced education and health-related academic backgrounds. Additionally, completion of BLS or first aid training was positively correlated with increased CPR knowledge. These findings highlight the importance of incorporating CPR training into educational curricula and public health programs to enhance awareness and improve bystander response and survival rates in out-of-hospital cardiac arrests.

## Introduction

According to the World Health Organization (WHO), cardiovascular diseases are the leading cause of death globally, accounting for approximately 17 million deaths annually, with sudden cardiac arrest (SCA) as a significant contributor to this alarming statistic [1]. SCA, the abrupt loss of heart function, is a critical medical emergency that requires immediate attention, as survival is highly dependent on timely interventions such as cardiopulmonary resuscitation (CPR) [2]. CPR, if initiated promptly and effectively, has been shown to restore partial circulation to vital organs, significantly improving outcomes [3].

Early recognition of SCA and immediate initiation of CPR and defibrillation within the first 5 minutes can increase survival rates to as high as 50%-70% [4]. These lifesaving interventions highlight the importance of adhering to standardized CPR protocols. International bodies, including the American Heart Association (AHA) and the International Liaison Committee on Resuscitation (ILCOR), review and update CPR guidelines every five years to reflect advances in resuscitation science [5]. The most recent updates emphasize bystander involvement, effective chest compressions, and defibrillation as critical components of successful resuscitation [5].

A comprehensive review of 21,623 resuscitation-related articles, published in 2022, highlighted significant disparities in bystander CPR performance and its impact on survival rates across different regions [6]. The study identified public awareness and training as key factors influencing bystander intervention, emphasizing the need for community education initiatives to improve outcomes globally [6]. Such findings align with global health priorities that aim to reduce preventable deaths from SCA through widespread awareness and training campaigns [7].

Saudi Arabia, with a growing population of approximately 36 million people, a significant proportion of whom are under 35 years old, offers a unique context for this study [8]. Public health efforts in the country increasingly focus on emergency care, making it essential to assess the population’s awareness of CPR. While CPR and basic life support (BLS) training are not yet fully integrated into the national school curriculum, efforts have been made to introduce basic life-saving skills through awareness campaigns and optional programs in some schools. Some initiatives target students under 15 years old through optional workshops and awareness programs, particularly in regions like Riyadh and Jeddah. These programs are often led by certified instructors from organizations such as the Saudi Heart Association (SHA) and aim to teach basic skills like CPR and automated external defibrillator (AED) use. However, most programs remain optional and are more prevalent at the university level, particularly in health-related fields such as medicine, nursing, and allied health sciences, where they are often included as part of the curriculum and practical training. Outside of health-related fields, exposure to CPR training varies and is typically provided through optional workshops or external organizations.

This study aims to evaluate the knowledge and awareness of CPR among the Saudi population, identify gaps, and propose targeted health interventions, including education campaigns and training programs, to enhance community preparedness in emergencies and improve survival rates for SCA victims.

## Materials and methods

Study design and study population

A cross-sectional observational study was conducted from May 2023 to November 2023. The study population involved citizens and residents aged 18 years and older from the Central, Eastern, and Western regions of Saudi Arabia to ensure a diverse range of cultural and social backgrounds. The sample size was 4932 and was determined using the Raosoft sample size calculator (Raosoft, Inc., Seattle, WA). This number represents approximately 0.014% of Saudi Arabia’s estimated population of 36 million. While this proportion may appear modest, the sample size exceeds the statistical requirements for robust and meaningful analysis. Based on a 95% confidence level, a 5% margin of error, and an estimated prevalence of 50%, the minimum required sample size would be 384 participants, or 1068 if the margin of error was reduced to 3%. Additionally, a power analysis designed to detect small effect sizes (0.1), with a significance level of 0.05 and a power of 0.8, indicates that at least 784 participants would be sufficient. Given these considerations, the sample size used in this study is significantly above these thresholds, ensuring precision, statistical power, and representativeness, thereby making it appropriate for assessing CPR knowledge, attitudes, and practices within the Saudi population.

Study questionnaire

Data were collected using an online, validated, closed-ended, structured questionnaire distributed via social media platforms, specifically WhatsApp and Telegram. The questionnaire used in this study was self-developed, and designed to assess the knowledge, attitudes, and practices related to CPR. The questions were formulated based on the American Heart Association (AHA) fundamental CPR concepts guidelines, which emphasize the importance of CPR knowledge and public education [9]. The questionnaire included questions related to four distinct areas. The first section included questions related to the demographics of the respondents, including age, gender, nationality, education, academic specialization, and employment status. Some questions were included to assess the respondents’ BLS training, whether they have witnessed any situations related to SCA, and their actions. Another set of questions was included to assess respondents’ knowledge regarding SCA, CPR, and BLS. In addition, questions were included to assess how the respondents’ actions would differ based on whether the person experiencing SCA was a stranger or someone close (friend or relative).

Questionnaire scoring

The questionnaire scoring was performed for the 13 knowledge questions. The correct responses for the included questions have been presented in the Appendix. Respondents were awarded one point for each correct answer. Thus, a maximum score of 13 was possible.

Statistical analysis

Statistical analysis was performed using R v 4.3 (R Foundation for Statistical Computing, Vienna, Austria). Counts and percentages were used to summarize categorical variables. The mean ± standard deviation (SD) and the median/interquartile range (IQR) were used to summarize continuous normal and non-normal variables, respectively. The chi-square test of independence was used to assess the association between categorical variables. The unpaired t-test and Mann-Whitney test were used to compare continuous normal and non-normal variables. Hypothesis testing was performed at a 5% level of significance.

## Results

A total of 4932 respondents completed the questionnaire. The demographic characteristics of the respondents are shown in Table 1. The gender distribution was predominantly female (76.5%, n = 3775), compared to 23.5% (n = 1157) males. The age distribution was skewed toward the younger population, with 59.1% (n = 2914) aged between 18 and 30 years. The educational level was predominantly university-level (68.8%, n = 3391). The academic specialization was diverse, with the largest group being healthcare practitioners (47.1%, n = 1615). The study found that 44.7% (n = 2203) of respondents had received BLS or first aid training, while 55.3% (n = 2729) had not. This points to almost half of the sample having formal emergency response training. Results suggest a trend toward higher knowledge levels. The average knowledge score among the respondents was 7.17 ± 2.37, indicating that respondents answered slightly more than half of the questions correctly. 

**Table 1:** Descriptive statistics for the study sample

Variable	Value (n = 4932)
Gender:	
Female	3775 (76.5%)
Male	1157 (23.5%)
Age:	
<18	510 (10.3%)
18-30	2914 (59.1%)
30-45	1008 (20.4%)
>45	500 (10.1%)
Nationality:	
Non-Saudi	489 (9.91%)
Saudi	4443 (90.1%)
Educational level:	
Elementary	33 (0.67%)
Intermediate	98 (1.99%)
Secondary	1044 (21.2%)
University	3391 (68.8%)
Higher education	366 (7.42%)
Academic specialization:	
Agriculture	43 (1.25%)
Business	471 (13.7%)
Computer science	290 (8.46%)
Customer service	63 (1.84%)
Engineering/Technology	202 (5.89%)
Healthcare practitioner	1615 (47.1%)
Journalism/Media communication	51 (1.49%)
Military	108 (3.15%)
Teacher/Professor	585 (17.1%)
Employment status:	
Non-employee	891 (18.1%)
Retired	132 (2.68%)
Student	2394 (48.5%)
Worker (different from academic major)	388 (7.87%)
Worker (same as academic major)	1127 (22.9%)

Knowledge scoring

**Figure 1:**
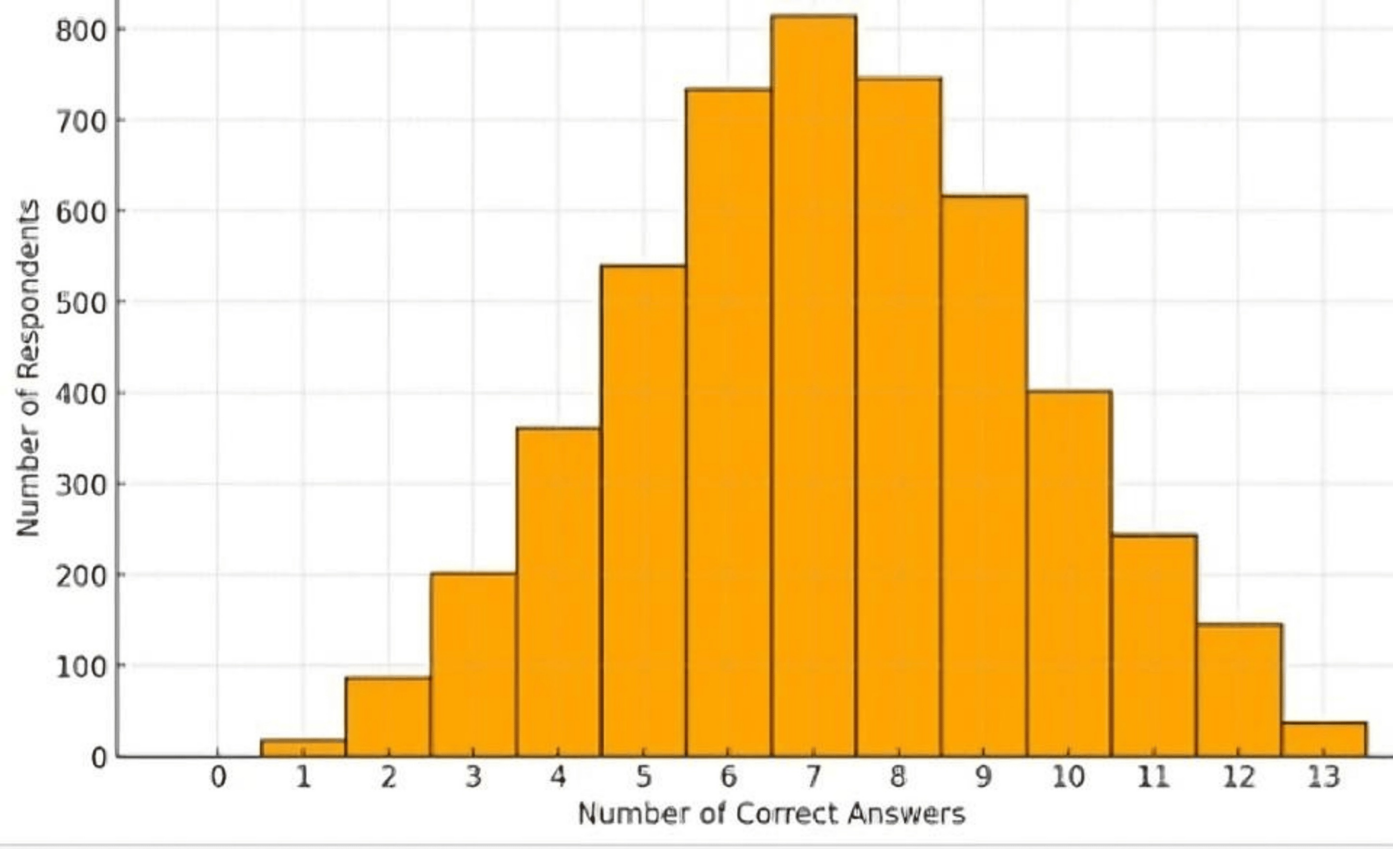
Distribution of knowledge score

Results suggest a trend toward higher knowledge levels. The average knowledge score among the respondents was 7.17 ± 2.37, indicating that respondents answered slightly more than half of the questions correctly. Looking at the quartiles, 25% of respondents scored six or fewer correct answers, and 75% scored nine or fewer. This distribution indicates that many respondents fall within the middle to higher scores range. These findings depict a group with varied yet generally higher knowledge levels in first aid and emergency response compared to the previous analysis. The concentration of scores toward the higher end suggests a reasonable level of awareness, likely influenced by the updated scoring criteria. However, there is still room for improvement in knowledge and emergency preparedness among the surveyed group (Figure 1).

**Figure 2:**
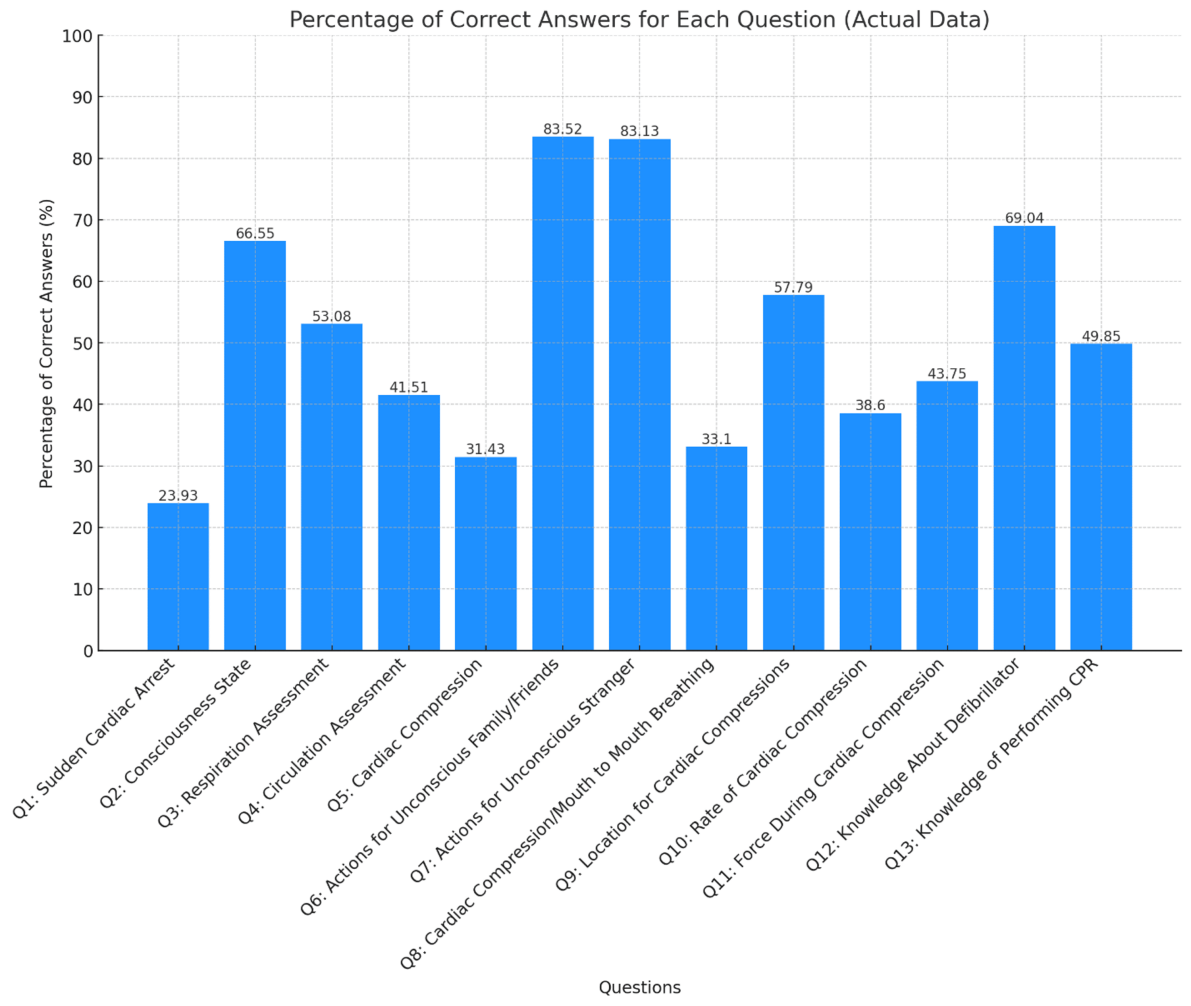
Percentage of correct answers for the included questions

Only two questions had a high percentage (>75%) of correct answers (Q6 and Q7). There was a noticeable variation in the percentage of correct answers across different questions, reflecting the respondents’ differing knowledge or understanding of each topic. Details regarding the percentage of correct answers are shown in Supplementary Table 6. Certain areas, such as “Knowledge About Defibrillator” and “Consciousness State Assessment,” show high percentages of correct answers (69.04% and 66.55%, respectively), indicating substantial knowledge in these areas. Questions like “Absence of Respiration Assessment” and “Location for Administering Cardiac Compressions” had moderate correctness percentages (53.08% and 57.79%, respectively) (Figure 2).

A majority of respondents (83.0%, n = 4093) reported never having witnessed a sudden death. Among those who had, witnessing the sudden death of a stranger was reported by 5.07% (n = 250), of a family member by 8.33% (n = 411), and of a friend or acquaintance by 3.61% (n = 178). This indicates a relatively limited direct exposure to sudden death situations among the sample population. Of the 899 respondents who have witnessed a sudden death, 21.5% (n = 193) began giving cardiac compressions, and 14.0% (n = 126) performed both cardiac compression and mouth-to-mouth breathing (CPR). Only 11.1% (n = 100) called an ambulance, while 17.5% (n = 157) performed mouth-to-mouth breathing. Notably, 10.7% (n = 96) did not witness such an event, 6.79% (n = 61) just watched and left, and 18.5% (n = 166) instructed someone to call for help. These responses highlight a diverse range of actions, with a significant portion initiating some form of BLS intervention. The study found that 44.7% (n = 2203) of respondents had received BLS or first aid training, while 55.3% (n = 2729) had not. Regarding specific BLS skills, 24.7% (n = 1217) of respondents reported knowing cardiac compression, 11.6% (n = 572) could open the airway, 28.8% (n = 1422) knew both ventilation and cardiac compression, and 4.79% (n = 236) were familiar with ventilation and mouth-to-mouth breathing. However, 26.9% (n = 1328) did not know any BLS skills. Most respondents (62.0%, n = 3058) were unaware of where to find an AED or pacemaker, while 38.0% (n = 1874) had this knowledge (Table 2).

**Table 2:** Community experience and response in sudden cardiac emergencies and BLS knowledge BLS, basic life support.

Item	Total respondents (N = 4932)
Ever witnessed a sudden death:	
A stranger	250 (5.07%)
I have not seen this	4093 (83.0%)
Somebody from my family	411 (8.33%)
Somebody from my friends or acquaintances	178 (3.61%)
Action during the event:	
I began to give cardiac compression	193 (21.5%)
I gave both cardiac compression and conducted mouth-to-mouth breathing (I gave CPR)	126 (14.0%)
I called an ambulance	100 (11.1%)
I conducted mouth-to-mouth breathing	157 (17.5%)
I have not seen this	96 (10.7%)
I just watched and left	61 (6.79%)
I told somebody to call for help	166 (18.5%)
BLS/First aid training:	
No	2729 (55.3%)
Yes	2203 (44.7%)
BLS skills:	
Cardiac compression	1217 (24.7%)
I can control respiration	157 (3.18%)
I can open the airway	572 (11.6%)
I do not know	1328 (26.9%)
Ventilation and mouth-to-mouth breathing	236 (4.79%)
Ventilation and cardiac compression	1422 (28.8%)
Location of an automated external defibrillator or “pacemaker”	
I do not know	3058 (62.0%)
Yes	1874 (38.0%)

**Figure 3:**
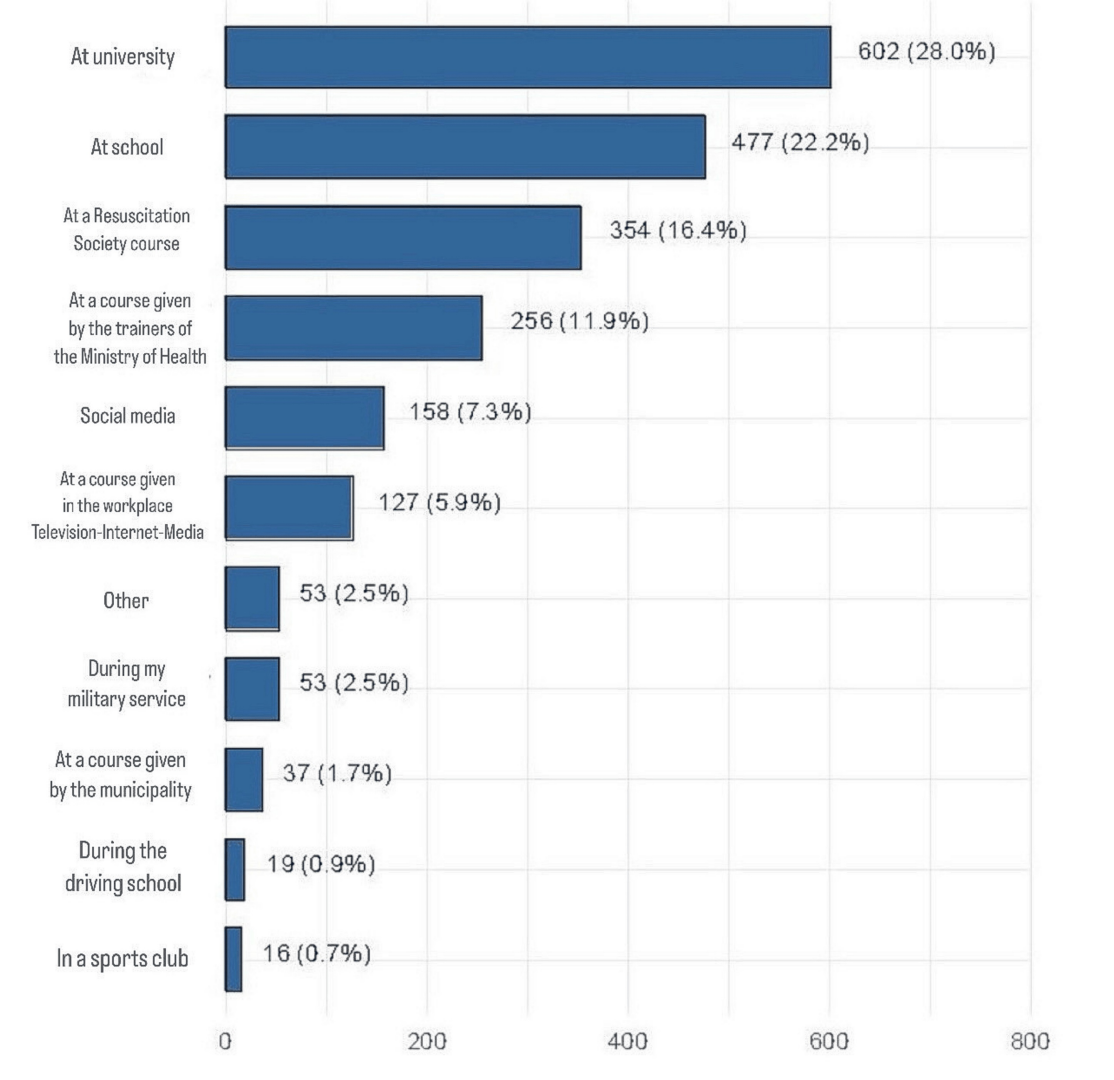
Source of BLS/first aid training BLS, basic life support.

Figure 3 shows the source of BLS/first aid training among respondents who reported receiving such training. The most common source of training was university (28%, n = 602), followed by the school (22.2%, n = 477), resuscitation society courses (16.4%, n = 354), and courses provided by trainers in the ministry of health (11.9%, n = 256).

**Figure 4:**
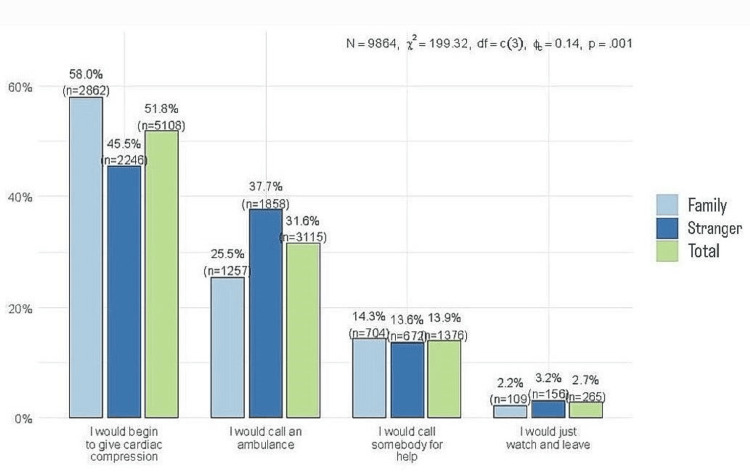
Comparative analysis of immediate responses to sudden cardiac death Analysis was performed using the chi-square test of independence.

The most common course of action was performing cardiac compression (51.8%, n = 5108), followed by calling an ambulance (31.6%, n = 3115). Notably, there was a statistically significant difference based on the victim (P = 0.001). Respondents were more likely to perform cardiac compressions when the person who experienced the event was a family member/friend (58%, n = 2862) compared to when he was a stranger (45.5%, n = 2246). Consequently, respondents were more likely to call an ambulance if the person at risk was a stranger (37.7%, n = 1858) (Figure 4).

**Figure 5:**
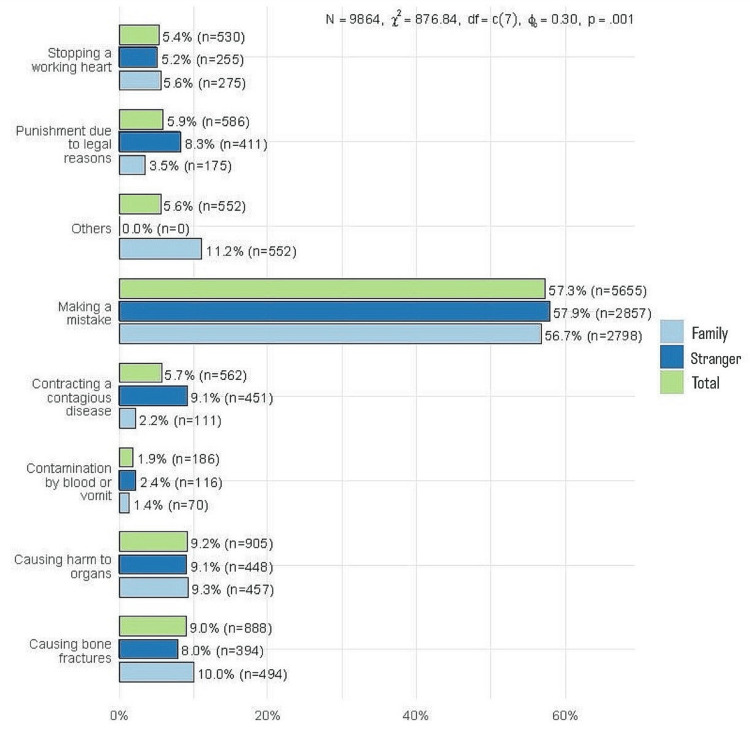
Comparative analysis of concerns related to applying CPR CPR, cardiopulmonary resuscitation.

The most common concern for applying CPR was making a mistake (57.3%, n = 5655). The percentage of respondents followed by contracting a contagious disease (5.7%, n = 562) and punishment due to legal issues (5.9%, n =586). Of note, these two concerns were more frequently reported when the person at risk was a stranger (9.1%, n = 451 and 8.3%, n = 411, respectively) than when the person was a friend/relative (2.2%, n = 111 and 3.5%, n = 175, respectively) (Figure 5).

**Figure 6:**
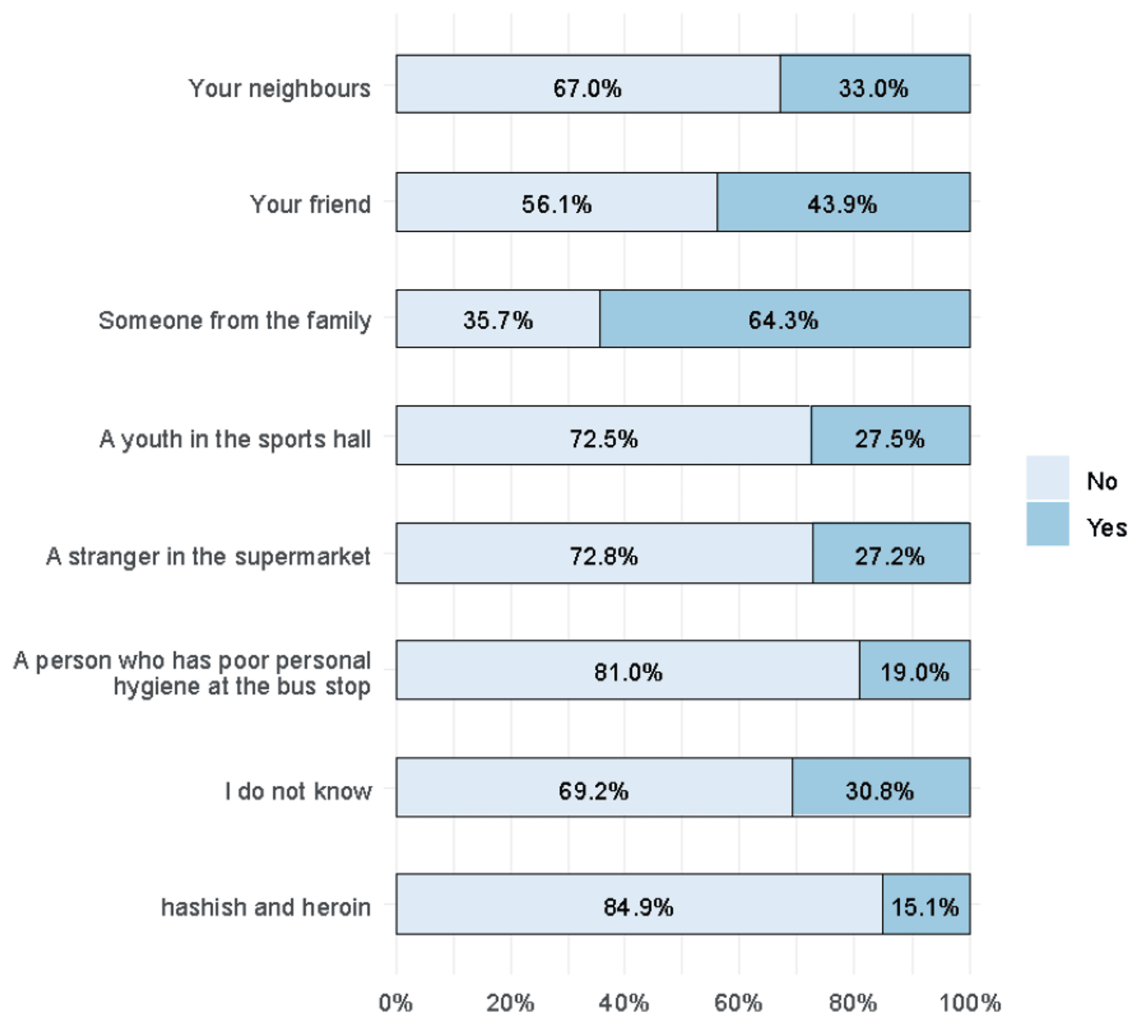
Recipients of CPR in sudden cardiac arrest CPR, cardiopulmonary resuscitation.

Two-thirds of the respondents chose someone from the family as a preferred recipient for CPR (64.3%). Other recipients were friends (43.9%) and neighbors (33%). People with poor personal hygiene (19%), hashish, and heroin users were the least preferred (15.1%) (Figure 6).

Factors associated with knowledge score

**Figure 7:**
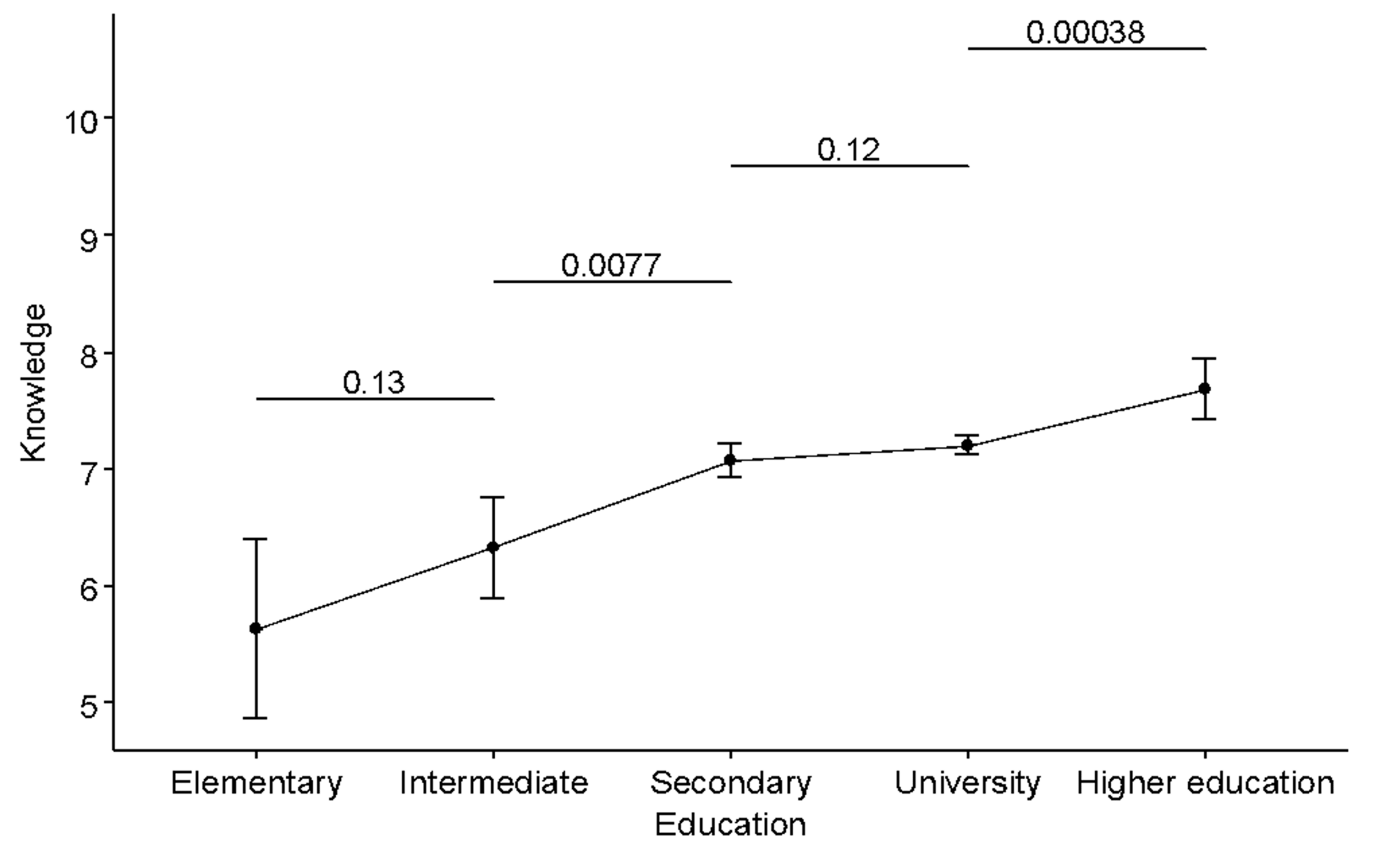
Association between education and knowledge score Analysis was performed using an unpaired t-test with Tukey adjustment.

As shown in Table 3, respondents with elementary education (n = 33) had an average knowledge score of 5.64 ± 2.16, which was significantly different from the average knowledge score in both secondary and university groups (Group 2), as well as the higher education group (Group 3). This suggests that this group’s average knowledge level is significantly lower than the average knowledge score of respondents with higher educational levels. Respondents with intermediate education (n = 98) have an average score of 6.33 ± 2.20, which was also significantly lower than the average in respondents with secondary, university, and higher education. Respondents with secondary and university education (Group 2) have average knowledge scores of 7.08 ± 2.35 and 7.20 ± 2.35, respectively, and were not significantly different (P > 0.05). However, their average score significantly differed from respondents with elementary and intermediate education (P < 0.05). Respondents with higher education (n = 366) had an average knowledge score of 7.68 ± 2.5, which was significantly different from the average knowledge score in all other groups (P < 0.05). These results are illustrated in Figure 7. 

**Table 3:** Factors associated with knowledge regarding CPR and BLS Data were summarized using the mean and standard deviation. Analysis was performed using one-way ANOVA. Post hoc pairwise comparisons were performed using an unpaired t-test with Tukey adjustment. Groups with similar numbers have significantly different means at the 0.05 levels. CPR, cardiopulmonary resuscitation; BLS, basic life support; ANOVA, analysis of variance.

Variable	Level	n	Mean	SD	F	P_ANOVA_	Post hoc
Academic specialization	Agriculture	43	6.35	2.18	78.32	<0.001	12
	Business	471	6.46	2.07			1
	Computer science	290	6.79	2.06			12
	Customer service	63	5.98	2.06			1
	Engineering/Technology	202	7.25	2.05			2
	Healthcare practitioner	1615	8.40	2.38			3
	Journalism/Media communication	51	6.55	1.81			12
	Military	108	6.55	2.10			12
	Teacher/Professor	585	6.34	2.07			1
Age	<18	510	6.85	2.04	48.59	<0.001	1
	18-30	2914	7.50	2.37			2
	30-45	1008	6.79	2.42			1
	>45	500	6.43	2.25			3
Education	Elementary	33	5.64	2.16	11.48	<0.001	1
	Intermediate	98	6.33	2.20	11.48		1
	Secondary	1044	7.08	2.35	11.48		2
	University	3391	7.20	2.35	11.48		2
	Higher education	366	7.68	2.50	11.48		3
Employment status	Non-employee	891	6.38	2.18	50.74	<0.001	1
	Retired	132	6.53	2.21	50.74		1
	Worker (different from academic major)	388	6.66	2.26	50.74		1
	Student	2394	7.57	2.32	50.74		2
	Worker (same as academic major)	1127	7.25	2.46	50.74		3
Gender	Female	3775	7.17	2.39	0.07	0.78	
	Male	1157	7.20	2.31			
Nationality	Non-Saudi	489	7.17	2.49	0.00	0.95	
	Saudi	4443	7.18	2.35			
BLS training	Yes	2203	8.34	2.22	1189	<0.001	
	No	2729	6.24	2.05			

Respondents aged under 18 years (n = 510) had an average knowledge score of 6.85 ± 2.04, which was significantly different from the 18-30 age group (Group 2) but not significantly different from the 30-45 age group (Group 1). This indicates that the knowledge level among the youngest respondents is relatively lower compared to young adults but similar to middle-aged adults. For the age group of 18-30 years (n = 2914), the average knowledge score was 7.50 ± 2.37. This group’s score was significantly higher than the <18 years group and the 30-45 years group, suggesting that young adults in this age range have a higher average knowledge level than younger and middle-aged groups. Respondents aged 30-45 years (n = 1008) had an average score of 6.79 ± 2.42, which was not significantly different from the <18 years group but significantly lower than the 18-30 years group. This implies a similar level of knowledge between the youngest and the middle-aged groups, both lower than the young adult group. Finally, respondents over 45 years (n = 500) had an average knowledge score of 6.43 ± 2.25, significantly different from all other age groups. The lowest average score in this group suggests a decrease in the average knowledge level compared to the younger age groups, particularly the 18-30 years group. 

This analysis reveals a nuanced understanding of the knowledge levels across different age groups, showing that young adults aged 18-30 have the highest average knowledge score, while there is a notable decrease in the score for the oldest age group (>45 years). Respondents who were non-employees (n = 891) had an average knowledge score of 6.38 ± 2.18, significantly different from students and workers in the same academic major (Groups 2 and 3). This suggests that non-employees, on average, had a lower level of knowledge than these groups. Similarly, retired individuals (n = 132) and workers in roles different from their academic major (n = 388) had average knowledge scores of 6.53 ± 2.21 and 6.66 ± 2.26, respectively. 

These groups, like non-employees, were not significantly different from each other in their average knowledge scores (all in Group 1) but had significantly lower scores compared to students and workers in the same academic major. Students (n = 2394), with an average knowledge score of 7.57 ± 2.32, and workers in the same academic major as their field of study (n = 1127), with an average score of 7.25 ± 2.46, were in different groups (Groups 2 and 3, respectively, P < 0.05). Students had the highest average knowledge score, significantly different and higher than non-employees, retired individuals, and workers in different fields (P < 0.05 for all pairwise comparisons). Workers in the same academic major also had a higher average score than the first three groups, but their scores significantly differed from those of the students (P < 0.05). 

Among the various academic specializations, respondents with a background in health care (n = 1615) had a notably higher average knowledge score of 8.40 ± 2.38. This score was significantly different from all other academic specializations, as indicated by its unique grouping (Group 3) in the post hoc analysis (P < 0.05 compared to all other specializations). The considerable difference in the average score for healthcare practitioners suggests a substantially higher level of knowledge in the studied variable compared to other specializations. For context, specializations such as Agriculture, Business, Computer Science, Customer Service, Journalism/Media Communication, Military, and Teacher/Professor were grouped together (Groups 1 and 2), with average scores ranging from 5.98 to 6.79. These scores were significantly lower than those of the healthcare practitioners (P < 0.05). Engineering/Technology also stood out with a higher average score (7.25), but it was still notably lower than the score for healthcare practitioners. 

Respondents who had received BLS training (n = 2203) demonstrated a significantly higher average knowledge score of 8.34 ± 2.22 compared to those who had not received BLS training (n = 2729), who had an average score of 6.24 ± 2.05 (P < 0.001).

One-way ANOVA showed that education was significantly associated with knowledge score (P < 0.001). There was a statistically significant increasing linear trend (P < 0.001). Post hoc pairwise comparisons showed that the average knowledge score was significantly higher in respondents with higher education than in respondents with university education (P = 0.00038). The average knowledge score was higher in respondents with secondary education than in patients with intermediate education (P = 0.0077) (Figure 7).

There were notable variations in the average knowledge score based on different health conditions. Individuals without cardiac disease (n = 4752) had an average knowledge score of 7.19 ± 2.37, similar to those with cardiac disease (n = 79), who scored 6.85 ± 2.44, with no significant difference (P = 0.20). Those without diabetes (n = 4621) had an average score of 7.20 ± 2.38, contrasting with diabetic individuals (n = 210), who had a lower average score of 6.79 ± 2.24 (P = 0.01), indicating a significant difference. In the case of hypertension, respondents without it (n = 4644) scored 7.21 ± 2.36 on average, whereas those with hypertension (n = 187) had a lower average score of 6.47 ± 2.46, a significant difference (P < 0.001). For those without any medical condition (n = 500), the average score was 6.93 ± 2.36, in contrast to 7.21 ± 2.37 for those with a condition (n = 4331), showing a significant difference (P = 0.01). However, respiratory diseases and other conditions did not show a significant impact on knowledge scores (P = 0.83 and P = 0.61, respectively), suggesting that these specific comorbidities may not significantly affect the knowledge levels in the studied variable (Table 4).

**Table 4:** Association between medical condition and knowledge score Data were summarized using the mean and standard deviation. Analysis was performed using an unpaired t-test.

Comorbidity	Level	n	Mean	SD	F	P
Cardiac disease	No	4752	7.19	2.37	1.63	0.20
	Yes	79	6.85	2.44		
Diabetes	No	4621	7.20	2.38	6.13	0.01
	Yes	210	6.79	2.24		
Hypertension	No	4644	7.21	2.36	17.79	<0.001
	Yes	187	6.47	2.46		
No medical condition	No	500	6.93	2.36	6.32	0.01
	Yes	4331	7.21	2.37		
Respiratory disease	No	4681	7.18	2.37	0.04	0.83
	Yes	150	7.23	2.31		
Other condition	No	4798	7.18	2.37	0.26	0.61
	Yes	134	7.28	2.36		

## Discussion

BLS is essential for maintaining an airway, supporting breathing, and ensuring circulation without the use of advanced medical equipment. This foundational emergency procedure is crucial for increasing survival rates during cardiac and respiratory emergencies. Studies indicate that effective BLS performed by bystanders significantly enhances the chances of survival in out-of-hospital cardiac arrest (OHCA) scenarios. For instance, BLS has been shown to double the number of people who survive the period before hospital admission when initiated promptly by a bystander [10]. Bystander CPR has been associated with improved survival rates and better neurological outcomes in both adults and children following OHCA [11]. Another study highlighted that effective bystander BLS can prevent the deterioration of ventricular fibrillation, thus improving the chances of successful defibrillation and overall survival [12]. 

Despite the importance of BLS, there is a notable gap in knowledge and training among the general population [13]. The low average knowledge score, particularly among those under 30 years of age with a university education, is concerning, as it suggests that even a relatively educated demographic lacks sufficient BLS awareness. This deficiency can significantly affect survival rates during cardiac or respiratory emergencies. Although a large portion of individuals expressed willingness to intervene in emergencies, with 21.5% initiating chest compressions and 14% providing both compressions and mouth-to-mouth breathing, the overall level of preparedness remains inadequate. This discrepancy highlights a critical need for improved BLS education and training within the community, especially in light of the clear gap between willingness and actual action during emergencies [14].

Several studies have highlighted the widespread lack of adequate BLS knowledge among various populations. For instance, a study in Portugal found that only 15.3% of the general public scored above 70% on BLS knowledge assessments, with less than 30% having received any formal BLS training [15]. Similarly, a study among a Swiss sample revealed that 20% had never received any BLS training, and a significant portion lacked basic knowledge such as the emergency phone number or the proper execution of CPR [16].

In Jeddah, a study revealed that the average BLS knowledge score was only 5.3 out of 15, highlighting a substantial knowledge gap [17]. Similarly, research among Saudi women's university students showed that 87.9% had very poor BLS knowledge scores [18]. In addition, another study found that the knowledge regarding BLS in the general non-medical population in Jeddah was below average [19]. These findings align with results from the current study, which included mainly females, and indicate that the majority of the general population in Saudi Arabia had only a moderate level of BLS knowledge, with many lacking formal training. This widespread lack of BLS knowledge underscores the need for comprehensive and accessible BLS training programs to improve public preparedness and response capabilities during emergencies. The findings underscore the urgent need for comprehensive and regular BLS training programs to enhance public preparedness and response capabilities in medical emergencies. 

Moreover, the study conveyed some worrying findings. For example, respondents with a medical background have an average knowledge score of 8.4, which although higher than that in the general population was still considerably low for individuals expected to be proficient in life-saving techniques. This gap highlights the need for enhanced training and education among medical professionals. Research suggests that medical students who have completed the theoretical courses and training programs should have acquired a comprehensive understanding of CPR. However, the reality is that they did not meet the anticipated results [20]. 

Implementing mandatory, periodic CPR training sessions and refresher courses, utilizing advanced simulation techniques, incorporating CPR training into continuous professional development programs, and increasing public awareness about the importance of CPR can significantly improve the proficiency of healthcare professionals in performing CPR. Regular practice and realistic scenarios are crucial for retaining CPR knowledge and skills, while continuous assessments and certifications can ensure healthcare workers stay updated with the latest guidelines and techniques. By addressing these areas through targeted educational and policy interventions, we can enhance the ability of healthcare professionals to perform CPR effectively, ultimately improving survival rates for cardiac arrest victims in the community. 

As previously noted, BLS training programs are not expected to achieve optimal results. Nonetheless, the effect of such programs is evident in the current study which showed that respondents with prior BLS training had significantly higher average scores (8.34) compared to those without (6.24). Similar findings were highlighted by González-Salvado who showed that integrating BLS training into cardiac rehabilitation programs improves patients' skill retention and confidence in performing BLS, thereby enhancing overall community preparedness. Therefore, widespread BLS training is essential for ensuring timely and effective emergency response, which can be the difference between life and death [21]. Additionally, BLS training has been found to significantly increase the motivation, knowledge, and skills of trainees in providing first aid during cardiac arrest incidents, as evidenced by a study conducted in Indonesia [22]. Based on these findings, the need for practical and regular BLS training should be emphasized, with a call for it to be included in the medical curriculum [23]. 

One-half of the respondents were healthcare professionals (HCPs) which explains the high proportion of respondents who received prior BLS training compared to other studies [24-26] with the percentage of respondents who reported receiving BLS training ranging from 3% to 25%. In contrast, the rate observed in the current study was still lower than the rates reported in developed countries. In the USA study which included 9022 individuals, 83% reported participating in one or more CPR training classes [27]. Out of the 1076 respondents in Australia, 56% stated that they had received CPR training before [28]. The disparity in CPR training rates between the current study and others may be attributed to differences in how CPR training is distributed and promoted across various segments of the population. Variations in access to training programs, the channels used to deliver such education (e.g., schools, universities, healthcare organizations, or media platforms), and the frequency of these programs can significantly influence the overall level of CPR awareness and skills in the community. This highlights the importance of ensuring widespread and consistent dissemination of CPR education to improve preparedness and response in emergency situations. In the current study, the most common source of BLS/first aid training was universities (28%, n = 602), followed by schools (22.2%, n = 477) and resuscitation society courses (16.4%, n = 354) with only 7.3% (n = 158) and 5.9% (n = 127) attributing their knowledge to social media and other media sources such as television. Hence, it is evident that public health campaigns play a crucial role in spreading information and expertise among the general population, and there is an urgent need for such efforts in countries where understanding of CPR is insufficient. The government can further endorse CPR on social media and television, platforms that are widely utilized by individuals for accessing such information. 

The study revealed that the most common concern regarding the application of CPR was the fear of making a mistake, reported by 57.3% (n = 5655) of respondents. This fear can be a significant barrier to performing CPR, as individuals might hesitate to intervene in emergency situations due to the anxiety of causing harm or failing to execute the procedure correctly. Other barriers include a lack of training opportunities, fear of harm, and a lack of confidence [29]. These results are consistent with those reported in other studies [30]. Addressing this concern through comprehensive training programs that build confidence and competence in performing CPR is crucial. Practical training sessions and simulations can help individuals become more comfortable and proficient in their skills, thus reducing the fear of making mistakes [17]. 

Concerns about legal repercussions and contracting contagious diseases were also significant, particularly when helping strangers compared to family members or friends. This indicates that individuals are more likely to assist people they know due to lower perceived personal risks and legal consequences. These concerns can be mitigated by public education on Good Samaritan laws, which protect bystanders who provide emergency assistance, and by emphasizing the low risk of disease transmission when proper precautions are taken. Public health campaigns and legal reforms that offer clear protections and guidelines can encourage more people to act in emergencies without fear of legal or health-related repercussions [19]. By addressing these concerns through targeted education and supportive policies, we can enhance public willingness to perform CPR, thereby increasing the chances of survival for cardiac arrest victims in the community.

Limitations 

The study had several limitations. First, it relied on self-reported data, which could introduce bias due to inaccurate or dishonest responses from participants. The cross-sectional design limits the ability to establish causality between variables. Additionally, the sample may not be fully representative of the entire Saudi population, as it was limited to respondents available during the survey period. Approximately 50% (n = 1615) of the participants were HCPs from the total academic specializations, which may skew the results toward a higher level of knowledge. This overrepresentation of healthcare practitioners could lead to an inflated perception of CPR awareness in the broader population, as individuals in medical and health-related fields typically receive more comprehensive and frequent training in life-saving techniques compared to the general public. The study was conducted via an online survey, which introduces a sampling bias as it predominantly includes individuals with higher levels of knowledge and access to the internet. 

The study focused on basic knowledge and awareness, without assessing practical skills or long-term retention of BLS knowledge, and participants might have had difficulty accurately recalling past experiences related to BLS and CPR training or emergencies. Furthermore, the geographical limitation of the study, being conducted in specific regions, might not reflect the knowledge and awareness levels across different areas of Saudi Arabia. These limitations suggest areas for improvement in future research, such as employing longitudinal designs, expanding geographical coverage, including practical assessments of BLS skills, and ensuring a more representative sample.

## Conclusions

The level of knowledge and awareness regarding the effectiveness of CPR among participants in the current study was relatively high. However, the findings also revealed key factors that influenced this awareness, such as the educational level and academic specialization of the participants. Specifically, individuals with higher educational attainment and those in health-related fields demonstrated a greater understanding of CPR’s effectiveness. Additionally, the study found a direct positive relationship between the completion of BLS or first aid training courses and higher CPR knowledge levels.

Based on these findings, it is clear that integrating CPR training into educational curricula and public health programs is essential for raising awareness and expanding knowledge across various segments of society. While HCPs tend to have more advanced knowledge due to their specialized training, it is equally important to increase CPR training among the general population, particularly given the critical role bystanders play in improving survival rates for OHCAs. Furthermore, encouraging healthcare professionals to recommend CPR courses to the general public could serve as an effective strategy for enhancing community preparedness, thus potentially reducing the number of preventable deaths from cardiac emergencies.

Overall, these findings underscore the importance of integrating CPR education into formal training programs, alongside public awareness campaigns, to ensure that individuals of all educational backgrounds and professions have the knowledge and confidence to respond effectively during a cardiac emergency. This approach could ultimately contribute to improved survival rates and better outcomes for OHCA victims.

## Appendices

**Table 5:** Correct answers for knowledge questions

N	Question Topic	Correct Answer Criteria
1	Signs of Sudden Cardiac Arrest	Includes both “Loss of consciousness” and “Discontinuation of breathing.”
2	Consciousness State Assessment	Includes “No response when called” or “No response when touched.”
3	Absence of Respiration Assessment	Includes “Not steaming up a mirror placed in front of the mouth” or “Not having any respiratory movement.”
4	Circulation Assessment	Includes “Not feeling a pulse in the vessels of the neck.”
5	Definition of Cardiac Compression	Describes applying strong compression to the chest at certain intervals.
6	Actions for Unconscious Family/Friends with No Pulse	Includes calling an ambulance or starting CPR.
7	Actions for an Unconscious Stranger With No Pulse	Includes calling an ambulance or starting CPR.
8	Ratio of Cardiac Compression/Mouth-to-Mouth Breathing	The ratio is 30/2.
9	Location for Administering Cardiac Compressions	States the middle of the chest.
10	Rate of Cardiac Compression	Mentions at least 100 times per minute.
11	Force Applied During Cardiac Compression	Mentions moderate force, such that the rib cage moves down or enough that the rib cage moves down 5 to 6 cm.
12	Knowledge About Defibrillator	States that it is a device to restart a heart that has stopped beating.
13	Knowledge of Performing CPR on a Person With No Breathing or Pulse	Answer should be “Yes”

**Table 6:** Responses to knowledge questions

	Total respondents (N = 4932)
Consciousness state assessment:	
I do not know	720 (14.6%)
No response when called	2096 (42.5%)
No response when touched	1188 (24.1%)
Not moving at all	928 (18.8%)
Absence of respiration assessment:	
I do not know	551 (11.2%)
No air coming out from the mouth of the individual	1101 (22.3%)
Not having any respiratory movement	2099 (42.6%)
Not having any respiratory sound	661 (13.4%)
Not steaming up a mirror placed in front of the mouth of individual	520 (10.5%)
Circulation assessment:	
I do not know	1217 (24.7%)
Not feeling a pulse in the vessels of the arm	965 (19.6%)
Not feeling a pulse in the vessels of the neck	2050 (41.6%)
The lack of circulation signs	700 (14.2%)
Definition of cardiac compression:	
I have no idea	1400 (28.4%)
To apply compression directly to the heart opening the chest wall	554 (11.2%)
To apply strong compression to the chest at certain intervals (compress)	1553 (31.5%)
To scrub the chest at certain intervals	1087 (22.0%)
To scrub the heart directly opening the chest wall	338 (6.85%)
Actions for unconscious family/friends with no pulse:	
I would begin to give cardiac compression	2862 (58.0%)
I would call an ambulance	1257 (25.5%)
I would call somebody for help	704 (14.3%)
I would just watch and leave	109 (2.21%)
Actions for an unconscious stranger with no pulse:	
I would begin to give cardiac compression	2246 (45.5%)
I would call an ambulance	1858 (37.7%)
I would call somebody for help	672 (13.6%)
I would just watch and leave	156 (3.16%)
Knowledge of performing CPR on an a person with no breathing or pulse:	
No	2475 (50.2%)
Yes	2457 (49.8%)
Proper rate of cardiac compression/mouth-to-mouth breathing:	
15/2	2095 (42.5%)
30/2	1636 (33.2%)
5/1	1201 (24.4%)
Location for administering cardiac compressions:	
Lower part of the chest	768 (15.6%)
Middle of the chest	2852 (57.8%)
Other	357 (7.24%)
Upper part of the chest	955 (19.4%)
Rate of cardiac compression:	
At least 100 times per minute	1903 (38.6%)
At least 150 times per minute	723 (14.7%)
At least 50 times per minute	774 (15.7%)
I do not know	1532 (31.1%)
How much force must be applied during cardiac compression?	
As much force as possible	332 (6.73%)
Enough that the rib cage moves down 1 to 2 cm	1113 (22.6%)
High force, such that the rib cage moves down 6 to 10 cm	548 (11.1%)
Moderate force, such that the rib cage moves down 5 to 6 cm	2159 (43.8%)
Other	780 (15.8%)
Knowledge regarding defibrillator	
I have heard of it before but have not seen it	694 (14.1%)
I have never heard of it	508 (10.3%)
It is a device supporting respiration	315 (6.39%)
It is a device to restart a heart that has stopped working	3415 (69.2%)
